# Bread Wheat Biofortification for Grain Carotenoid Content by Inter-Specific Breeding

**DOI:** 10.3390/foods12071365

**Published:** 2023-03-23

**Authors:** María Dolores Requena-Ramírez, Cristina Rodríguez-Suárez, Carmen M. Ávila, Carmen Palomino, Dámaso Hornero-Méndez, Sergio G. Atienza

**Affiliations:** 1 Instituto de Agricultura Sostenible (CSIC), Alameda del Obispo, s/n, E-14004 Córdoba, Spain; mdrequena@ias.csic.es (M.D.R.-R.); crodriguez@ias.csic.es (C.R.-S.);; 2 Área Mejora y Biotecnología, IFAPA-Centro Alameda del Obispo, Apdo. 3092, E-14080 Córdoba, Spain; carmenm.avila@juntadeandalucia.es; 3 Department of Food Phytochemistry, Instituto de la Grasa (CSIC), Campus Universidad Pablo de Olavide, Edificio 46, Ctra de Utrera, Km 1, E-41013 Sevilla, Spain; hornero@ig.csic.es

**Keywords:** carotenoids, durum wheat, marker-assisted selection, Phytoene synthase, bread wheat, biofortification

## Abstract

Bread wheat has traditionally been selected for whitish derived flours. As a consequence, the current varieties carry carotenogenic alleles associated with low grain carotenoid. In contrast, high grain yellow pigment content (YPC) has been a major target in durum wheat programs since yellow colour is an important aesthetic factor for pasta production. Phytoene synthase 1 (*Psy1*) genes have an important role in the determination of the carotenoid content in wheat. In this work, we have transferred the genes *Psy1-A1* and *Psy1-B1* from durum to bread wheat by inter-specific hybridization in order to evaluate the combined effect of these genes for the improvement of grain carotenoid content, as well as the development of carotenoid-enriched bread wheat lines. Inter-specific breeding coupled with a MAS approach based on *Psy1-A1* and *Psy1-B1* alleles has allowed the development of bread wheat pre-breeding lines with enhanced grain carotenoid content (16–23% mean). These biofortified lines have the potential to become new varieties or to be used as recurrent parents in bread wheat breeding programs.

## 1. Introduction

Wheat is one of the most important crops in the world. Both bread wheat (*Triticum aestivum* L.), and durum wheat (*Triticum turgidum* (L.) ssp. *durum* (Desf.) Husn.) are considered to be staple foods. The former is adapted to a wider range of environments, while the latter is more adapted to specific areas such as the Mediterranean basin. Both species differ in their chromosome constitution, bread wheat being a hexaploid (2n = 6x = 42) with three genomes (A, B and D) and durum wheat a tetraploid species (2n = 4x = 28) with the A and B genomes.

Improving yield and disease resistance are primary targets for both species. However, breeding strategies for quality are different and, for some traits, even opposite. For instance, bread wheat breeding has focused on obtaining grains with whitish endosperm to meet the industry requirements for the production of white flours. On the other hand, high grain yellow pigment content (YPC) is a main target in durum wheat programs since yellow colour is an important aesthetic factor influencing consumer choice for pasta acquisition [1,2]. Consumers usually prefer a bright yellow coloured pasta, so products that do not match these requirements are refused. Consequently, breeding programs worldwide have focused on the development of varieties with enhanced YPC [3]. The high heritability of YPC [4,5] has benefited wheat breeding for this trait using colorimetric determinations (CIE, colour-space system with positive b* index providing a measure of the yellowness) due to their simplicity and cost. Alternatively, spectrophotometric determinations using the AACC-approved method 14–50 are also used, although this method is more expensive, slower and has higher technical requirements than the previous one. In addition, many efforts have been dedicated to the identification of the genetic bases controlling YPC either by the identification of QTL using bi-parental populations, or by the detection of marker-trait associations using diversity panels reviewed by [6] with new target regions for specific carotenoids being identified in recent expanded analyses of wheat biodiversity [7,8]. However, it is widely known that the main QTLs for YPC content in both durum and bread wheat are located in the long arm of chromosomes 7A and 7B [4,5,9,10,11,12,13]. 

Carotenoids are isoprenoids synthesized by all photosynthetic organisms, and they are essential for photo-protection and participate in light harvesting [14]. These pigments are responsible for the colours in the range between yellow and red in non-photosynthetic tissues, and they confer the YPC in wheat and related species such as tritordeum. There are two types of carotenoids, considering their chemical composition: carotenes, which are exclusively formed by carbon and hydrogen, and xanthophylls, that also contain oxygen. These pigments are precursors of important hormones such as abscisic acid (ABA) and strigolactones, which are related to growth regulation and responses against biotic and abiotic stresses [14]. In addition, they also play important roles in relation to human nutrition. In general, all carotenoids have an antioxidant nature, but they also have specific functions such as provitamin A activity, which is exclusive to carotenoids with unsubstituted β-rings [15]. Other carotenoids, such as lutein and zeaxanthin, accumulate in the eye macula and their consumption has been associated with the alleviation of age macular degeneration [16]. The AACC 14–50 method expresses the total amount of carotenoids as β-carotene equivalent units, i.e., it indicates the total amount of carotenoids considering that all of them are β-carotene [17]. In the case of wheat, this has induced errors for farmers and millers on certain occasions since they assume that durum wheat has a high quantity of β-carotene, when this compound is a minor carotenoid in durum wheat grain since lutein accounts for 90% or higher of the carotenoid pool. This has even led to the incorrect labelling of commercial pasta as having high β-carotene content. Since this is untrue, it can be considered as misleading to potential consumers and it could have serious commercial consequences. Therefore, breeding for carotenoid biofortification should be coupled with analytical techniques (i.e., chromatographic), allowing the individual determination of carotenoids to provide accurate and specific information to millers and consumers. 

Carotenoid biosynthesis is well known due to the importance of these compounds at the nutritional level. The first step of the carotenoid pathway is controlled by the Phytoene synthase. This enzyme condenses two molecules of geranylgeranyl pyrophosphate (GGPP) to produce a molecule of phytoene [18], and it is considered to be the limiting step for carotenoid biosynthesis [19,20]. Indeed, the development of Golden Rice 2 was founded on the hypothesis that PSY was the limiting step in carotenoid biosynthesis [21]. In grasses, there are three Psy genes (*Psy1*, *Psy2*, and *Psy3*) [22]. Studies in maize have shown that while *Psy1* is critical for carotenoid accumulation in the endosperm, the roles of *Psy2* and *Psy3* were negligible. Indeed, candidate gene approaches allowed the demonstration that *Psy1-A1* and *Psy1-B1* were the genes underlying the genetic variation of QTL for YPC identified in chromosomes 7A and 7B of durum wheat [9,10]. The importance of this gene has promoted the development of diagnostic markers for alleles of both *Psy1-A1* and *Psy1-B1* [23,24,25], useful for marker-assisted selection for higher carotenoid content.

The divergent breeding strategies for grain colour deployed in durum and bread wheat offer an interesting opportunity to ascertain the real effects of *Psy1* loci in carotenoid accumulation in wheat. Since bread wheat has been selected for a whitish endosperm, it usually carries alleles associated with low grain carotenoid content in *Psy1* and other carotenogenic genes with minor effects. Accordingly, the transference of *Psy1-A1* and *Psy1-B1* from durum wheat to bread wheat by inter-specific hybridization would allow the evaluation of the combined effect of these genes for the improvement of grain carotenoid content, as well as the development of bread wheat lines enriched for grain carotenoid content.

The aims of this work were (1) to determine the effectiveness of marker-assisted selection (MAS) based exclusively on *Psy1-A1* and *Psy1-B1* to increase grain carotenoid content in bread wheat; (2) the determination of the combined effect of alleles for high carotenoid content from both genes; (3) to develop pre-breeding bread wheat lines with enriched carotenoid content.

## 2. Materials and Methods

### 2.1. Plant Material and Field Testing

**Figure 1:**
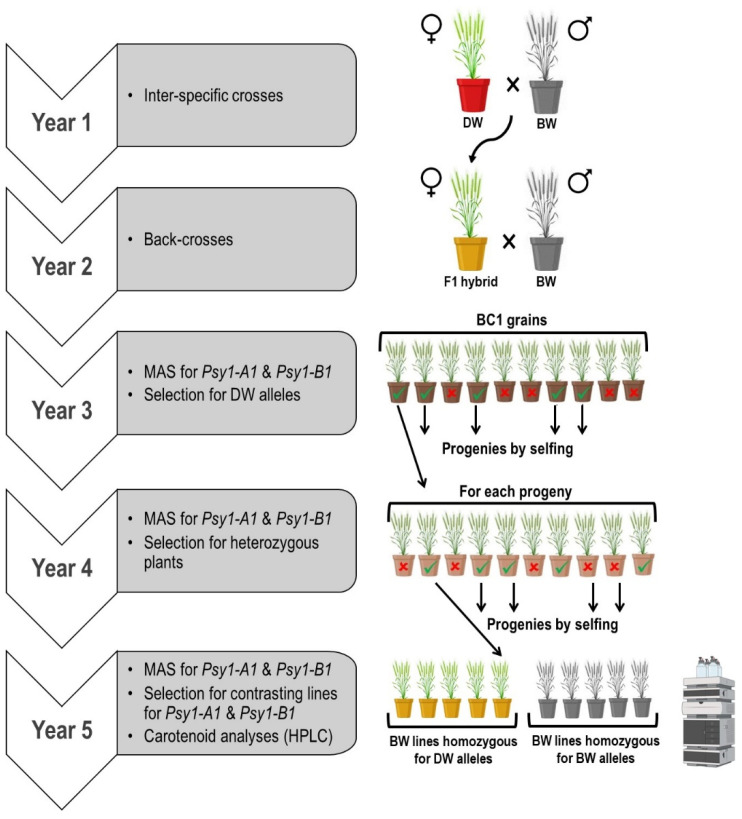
Crossing scheme and selection for the transference of *Psy1-A1* and *Psy1-B1* from durum wheat (DW) to bread wheat (BW) (Image created with Biorender.com).

A panel of durum wheat varieties and bread wheat (BW) advanced lines (kindly supplied by Dr. J.C. Sillero, IFAPA, Córdoba, Spain) were genotyped with markers for *Psy1-A1* and *Psy1-B1*, as described below. Two BW lines (12THES4515 and ES07H044SWC29) and the durum wheat (DW) elite variety ‘Kiko Nick’ differed in the alleles at both genes, and they were selected as parental lines. Two inter-specific crosses were performed: ‘Kiko Nick’ × 12THES4515 (referred to as cross KN × THES), and ‘Kiko Nick’ × ES07H044SWC29 (referred to as cross KN × C29). Subsequent plant material generation and selection were developed as described in Figure 1 under greenhouse conditions at IAS-CSIC (Córdoba, Spain). Briefly, ‘Kiko Nick’ was crossed as the maternal parent with each of the BW lines. The F_1_ hybrids were backcrossed with their respective BW parents. After this, BC_1_ grains were sown and genotyped with markers for *Psy1-A1* and *Psy1-B1* genes, as described below. Plants carrying DW alleles simultaneously for both genes were selected and selfed. During the fourth year, heterozygous plants for both genes were identified by MAS and selfed. Progenies of these plants were genotyped at two leaf-stage at greenhouse for both *Psy1-A1* and *Psy1-B1* to identify contrasting siblings carrying either BW alleles or DW alleles for both genes. These plants were grown outdoors under an anti-bird netting structure and with an anti-weed net. Grain samples were independently harvested from each plant and used for carotenoid analysis.

### 2.2. DNA Isolation and Marker-Assisted Selection

Genomic DNA was isolated using the CTAB method according to [26], with slight modifications. The detailed protocol used in our lab is shown in [27]. Primers YP7A-2, YP7B-1 and YP7B-4 were used for the amplification of the candidate genes since they allowed discrimination between the parental lines used in this work, as resulted from preliminary work [24,25]. PCR amplifications were performed with MyTaqTM DNA polymerase (Bioline, London, UK) following the manufacturer’s instructions. PCR conditions were as reported by [24,25]. Amplification products were resolved in agarose gels and visualized using SafeviewTM Nucleic Acid Stain (NBS biologicals, Ltd., Cambridgeshire, UK). 

### 2.3. Extraction of Carotenoids and HPLC Analysis

Carotenoids pigments were extracted from wheat grains according to the method previously described in [27]. Briefly, 1 g of grain sample and 6 mL of HPLC grade acetone (containing 0.1% BHT) were milled in an oscillating ball mill Retsch Model MM400 (Retsch, Haan, Germany) with two stainless-steel balls (15 mm Ø) at 25 Hz for 1 min. The resulting slurry was placed in a centrifuge tube (15 mL) and centrifuged at 4500× *g* for 5 min at 4 °C. The acetone phase was transferred to another plastic centrifuge tube, and the solvent was evaporated under nitrogen stream. The concentrated residue containing the pigments was dissolved in 0.5 mL of HPLC grade acetone and stored at −30 °C until chromatographic analysis (HPLC). All samples were pre-cleaned by centrifugation at 13,000× *g* prior to the chromatographic analysis. All the steps of carotenoid extraction and analysis were performed under dimmed light to prevent carotenoid photo-degradation and isomerization.

The analysis of carotenoids was performed by HPLC as described in previous works [28,29]. The HPLC system consisted of a Waters e2695 Alliance chromatograph fitted with a Waters 2998 photodiode array detector, and controlled with Empower2 software (Waters Cromatografía, S.A., Barcelona, Spain). A reversed-phase column (Mediterranea SEA18, 3 μm, 20 × 0.46 cm; Teknokroma, Barcelona, Spain) was used. Pigment separation was achieved by a binary gradient elution, at a flow rate of 1 mL/min, using an initial composition of 75% acetone (HPLC-grade) and 25% deionized water (HPLC-grade), which was increased linearly to 95% acetone in 10 min, then raised to 100% in 2 min, and maintained at a constant. Detection was performed at 450 nm, and the online spectra were acquired in the 350–700 nm wavelength range.

Carotenoid quantification was performed using calibration curves (concentration range of 0.5–45 µg/mL) prepared from pure pigment standards. The concentration of (*Z*)-isomers of lutein was assessed by using the calibration curve for (*all-E*)-lutein. Similarly, lutein esters were determined as free lutein equivalents. All the analyses were carried out on the same day of the preparation of extracts and performed in duplicate. Data were expressed as µg/g fresh weight (µg/g fw).

### 2.4. Statistical Analyses

Analyses of variance for total carotenoid content and concentration of each compound were performed using Statistix version 10.0 (Analytical Software, Tallahassee, FL, USA). The use of *p* values followed the recommendations by [30], and thus they were reported as continuous quantities.

## 3. Results and Discussion

Before the initial crosses, the durum (DW) and bread wheat (BW) genotypes selected as parental lines were genotyped with the markers for *Psy1-A1* and *Psy1-B1* genes developed by [24]. The markers YP7A-2 and YP7B-4 allowed for distinguishing between the alleles of the DW and BW parental lines (Table 1).

The DW ‘Kiko Nick’ carried the *Psy1-A1d* allele as deduced from the 1001-bp fragment amplified with YP7A-2 primers [25]. Both BW lines amplified the 1684-bp fragment with the same primers. The *Psy1-A1d* allele found in ‘Kiko Nick’ is associated with high YPC content [24,25]. Regarding *Psy1-B1*, ‘Kiko Nick’ carries the *Psy1-B1e* allele as shown by the amplification of the dominant marker YP7B-4 [25]. This allele is also associated with high grain yellowness [25]. Therefore, we used the markers YP7A-2 and YP7B-4 for the development of the BW pre-breeding lines, as detailed in Figure 1.

**Figure 2:**
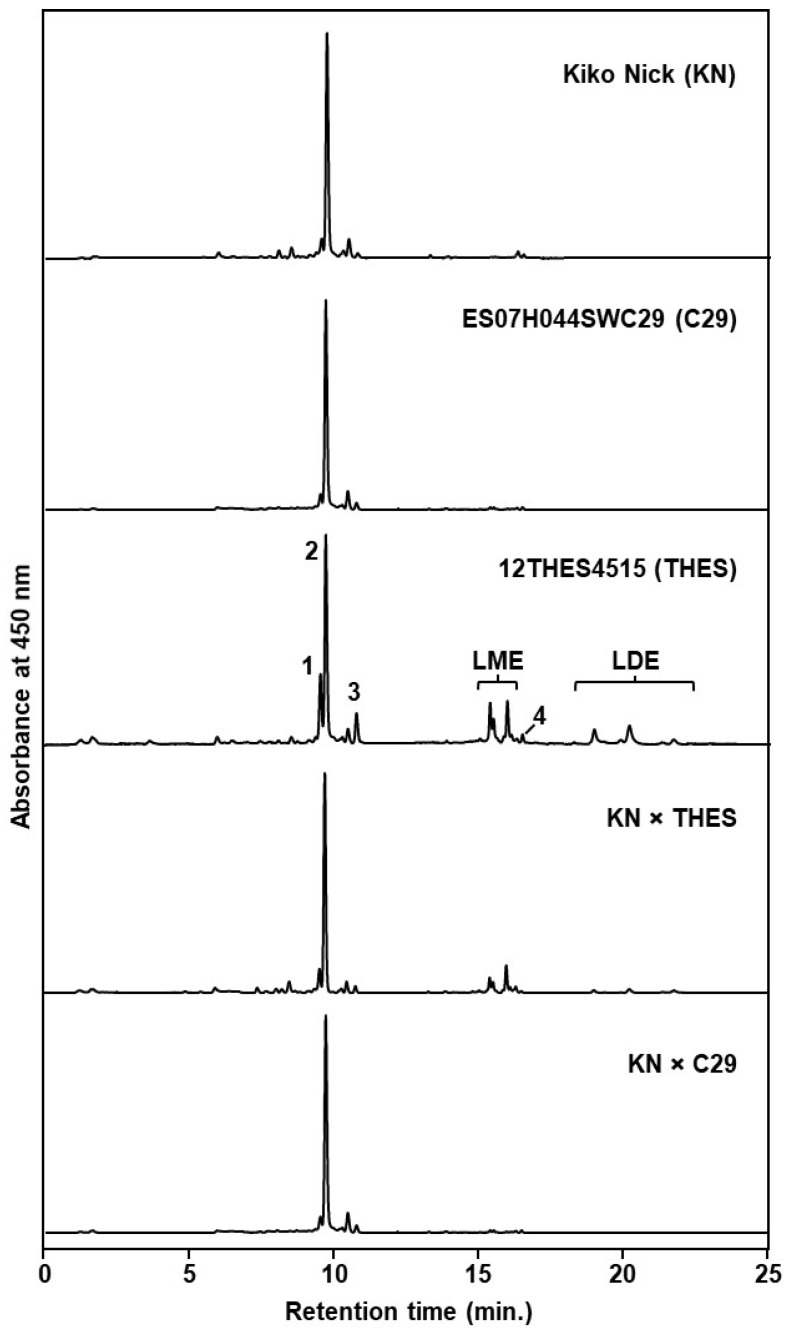
HPLC chromatograms corresponding to the grain carotenoid profile of the DW ‘Kiko Nick’, the BW breeding lines ES07H044SWC29, 12THES4515 and representative individuals of KN × THES and KN × C29 crosses. Peak identities: 1, (*all-E*)-zeaxanthin; 2, (*all-E*)-lutein; 3, (*Z*)-lutein isomers; 4, (*all-E*)-β-carotene; LME, lutein monoesters; LDE, lutein diesters. Detection wavelength was 450 nm.

The seeds obtained by selfing from the parental lines used for the initial crosses (year 1) were harvested and conserved at 4 °C. These seeds were later analysed for grain carotenoid content (Table 1, Figure 2). All three parental lines showed the typical carotenoid profile of wheat grains, as described in previous works [3,31,32,33,34]. 

Lutein was the main carotenoid, accounting for 85–95% of the total carotenoids with minor amounts of other carotenoids such as zeaxanthin and β-carotene (see Supplementary Table S1 for the detailed grain carotenoid profile). This is in agreement with previous knowledge, showing that lutein represents between 86 and 94% of the total carotenoids in wheat and related cereal grains [3,17,32,35]. The BW parental 12THES4515 had both lutein monoesters and lutein diesters. DW ‘Kiko Nick’ and BW ES07H044SWC29 showed very small amounts of lutein monoesters (<1.5% of total carotenoids), while lutein diesters were completely absent in both genotypes. Considering the low contribution of lutein esters to the total carotenoid pool, these lines can be classified as zero/low esters genotypes, as suggested by [31]. Indeed, the presence of low amounts of lutein monoesters in these lines can be explained by the time these samples were conserved at 4 °C, since lutein esterification can occur during seed storage [31,36,37]. On the other hand, lutein esters accounted for 35% of the total carotenoids in 12THES4515, which indicates the existence of an important esterification ability in this genotype.

The existence of lutein esters in 12THES4515 is relevant since esterification is considered a new target for carotenoid biofortification [14,38]. Lutein esters are more stable than free lutein during grain and flour storage [31,39,40,41], although the protective effect of esterification seems not to be relevant at very high temperatures, at boiling [42], extrusion or other high temperature regimes [43]. Nevertheless, lutein diesters are much more resistant to degradation than free lutein and lutein monoesters during the processing of white salted noodles [44]. Therefore, the improvement of carotenoid retention during grain storage due to esterification constitutes an interesting opportunity for the industrial sector, and thus the potential of this genetic trait is currently being explored in international efforts. Lutein esterification is common in bread wheat due to the D genome inherited from *Aegilops tauschii* Coss. [31]. Indeed, lutein esters were found in 40 out of 45 accessions of *Ae. tauschii* and in four accessions of *T. monococcum*, but no lutein esters were detected in any of the 17 accessions of durum wheat analysed. This has promoted the development of breeding programs to transfer the esterification ability to durum wheat, either by transferring the *XAT-7D* from common wheat [45] or the *XAT-7H^ch^* gene identified in the wild barley *H. chilense* [46] using an inter-specific breeding approach using a translocation line T7HchS·7A/B [47] carrying *XAT-7H^ch^* as donor parent [27]. However, a recent survey of 156 Spanish landraces allowed the identification of four accessions with high esterification ability [48], which opens new opportunities for the improvement of carotenoid esterification in durum wheat since they provide an additional source of esterification genes to the currently known *XAT-7D* and *XAT-7H^ch^*, which require recombination. 

In our case, the development of BW lines from the cross KN × THES, enriched for grain carotenoid content and with a high contribution of lutein esters, would accomplish a double biofortification strategy based on high carotenoid (lutein) content and stability.

After the crossing and selection steps detailed in Figure 1, BW lines carrying either DW or BW alleles simultaneously for both *Psy1-A1* and *Psy1-B1* alleles were obtained for each cross (year 5). Carotenoid determination was carried out in the harvested seeds of each plant (Supplementary Table S2). For each cross, the genotypes were grouped in two contrasting groups, either carrying DW or BW alleles for *Psy1-A1* and *Psy1-B1* genes (KN × C29-DW vs. KN × C29-BW and KN × THES-DW vs. KN × THES-BW). The mean values for carotenoid content were determined, as shown in Table 2. 

**Figure 3:**
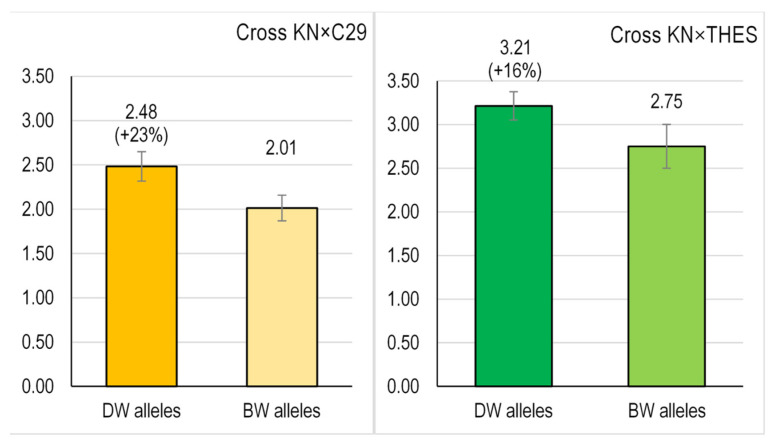
Phenotypic effect of the simultaneous presence of DW alleles for *Psy1-7A* and *Psy1-7B* on the total grain carotenoid content (µg/g fresh weight) of bread wheat lines obtained in this work. For each cross, bars represent the mean total carotenoid content ± SE.

For the cross KN × C29, we obtained thirteen breeding lines simultaneously carrying DW alleles for *Psy1-A1* and *Psy1-B1*, and seven lines carrying the alternative BW alleles (Supplementary Table S2). On average, the KN × C29-DW progeny had 2.48 µg/g grain carotenoid content, with a range between 1.05 and 3.10 µg/g (Figure 3, Supplementary Table S2). In contrast, the KN × C29-BW progeny showed a mean carotenoid content of 2.01 µg/g, with a range between 1.66 and 2.73 µg/g. Thus, the selection strategy for DW alleles increased the total grain carotenoid content by 23% (*p* = 0.080) (Figure 3). 

The same tendency was found in the KN × THES cross, where lineages carrying DW alleles (KN × THES-DW) outperformed those with BW alleles (KN × THES-BW) by 16% (mean values) (Figure 2). Indeed, the ten breeding lines with DW alleles yielded 3.21 µg/g of grain carotenoid mean content (range of 2.40–4.14 µg/g), whereas the seven lines with BW alleles showed a mean carotenoid content of 2.75 µg/g (*p* = 0.126) ranging from 1.66 to 3.52 (Figure 3). Considering both crosses, our results show an important effect of *Psy1* alleles for total carotenoid content, since a breeding strategy focused exclusively in two genes allowed an enhancement of carotenoid content between 16 and 23%.

In addition to this, it is important to note that all the breeding lines from the cross KN × THES produced lutein esters. Carotenoid esterification is controlled by *XAT-7D* in bread wheat [49], and thus this result was expected, considering the carotenoid profile of the parental line 12THES4515 (Table 1) and that all the breeding lines obtained carried the chromosome 7D. Bread wheat lines without lutein esters, such as Haruhikari, have been previously described [31] and the absence of lutein esters in ES07H044SWC29 is not surprising. 

Esterification is a mechanism for carotenoid accumulation and sequestration [50,51], and it has been related to the enhancement of carotenoid content [14]. Our results show that the progenies derived from the cross KN × THES had higher carotenoid content than those derived from the cross KN × C29 despite both ‘Kiko Nick’ and ES07H044SWC29 having almost double the carotenoid content of 12THES4515 (Table 1). These findings may be partly related to the esterification ability conferred by XAT-7D, that may improve carotenoid accumulation. However, the parental line 12THES4515 also carries a functional XAT-7D allele, but it has low carotenoid content. Thus, the higher carotenoid content of KN × THES progenies compared with KN × C29 suggests that other carotenoid-related genes are playing an important role in the cross KN × THES, in agreement with previous knowledge in wheat [6,8]. Indeed, a total of 124 significant marker-trait associations for lutein content were reported in common wheat, covering 18 out of 21 wheat chromosomes [7] and with six stable QTL on chromosomes 2AL, 2DS, 3BL, 3DL, 7AL and 7BS. Similar results have been obtained in durum wheat, with the identification of marker-trait associations for lutein and zeaxanthin grain content in several chromosomes [8]. These findings highlight the importance of other chromosome regions beyond *Psy1* genes for lutein accumulation in wheat.

In addition to the presence of the D genome, durum and bread wheat differ in a small quality trait gene package which determines the distinct technological properties necessary for their different end-use products: pasta or couscous, and bread, respectively. These few quality traits, mainly controlled by major genes, include the puroindoline loci (*Pin-a* and *Pin-b*) related to grain hardness, gliadins (Gli) and glutenins (Glu) loci, which determine gluten properties, and the *Psy1* gene, involved in carotenoid content/YPC reviewed by [52]. Through interspecific crosses, genes and/or alleles can be transferred in both directions to develop durum and bread wheat varieties with new or combined quality characteristics in order to obtain novel end-products, as demonstrated in this work. 

Previous attempts have allowed the development of high lutein BW lines (>6 µg/g of lutein) [31], but to the best of our knowledge these lines have not reach commercial status. Similarly, highly pigmented yellow grains are obtained in the synthetic wheat program at CIMMYT and routinely discarded, since traditional consumer preferences are oriented towards white flour. In this context, the pre-breeding BW lines with durum wheat *Psy1* alleles developed in this work constitute a good starting point towards the development of BW varieties with enhanced carotenoid content. In particular, pre-breeding lines with at least 3 µg/g of grain carotenoid content may cover an intermediate point between the current consumer preferences and the development of new products with creamy/yellow colour to fulfil new market demands. These pre-breeding lines outperform previous BW lines enriched in carotenoids [53] or other translocation lines developed throughout inter-specific breeding [34], where grain carotenoid content was 1.2 µg/g in the former case and below 2.1 µg/g in the latter, but they do not reach too much carotenoid content that may discourage potential consumers. 

However, it is important to note the existence of an emerging market for yellow-pigmented bread grains. For instance, the renewed interest in the nutritional aspects of foods has promoted the development of new breeding programs for einkorn (*Triticum monococcum* L. subsp. *monococcum*) [54], whose flour has a higher carotenoid content than bread wheat. Similarly, the commercial success of the new crop × *Tritordeum martinii* A. Pujadas has been partly associated with its higher carotenoid content [55]. Indeed, the high lutein content provides the golden colour of tritordeum-derived products, which is one of the most highlighted characteristics of this new cereal (https://www.tritordeum.com/?lang=en#whatis, accessed on 3 February 2023) since this trait is highly appreciated by the consumers. In this case, the availability of wheat lines biofortified for lutein content is useful for the commercialization of wheat-derived products with enhanced nutritional quality. Indeed, some authors refer to lutein as ‘the eye vitamin’ [7] due to its role in the alleviation of eye disorders such as age-macular degradation. Accordingly, marketing strategies for wheat grain and flours should focus on lutein instead of β-carotene, as has been wrongly done in certain cases.

## 4. Conclusions

*Psy1* genes have an important role in the determination of carotenoid content in wheat, although more attention should be paid to other genes of the carotenoid pathway. Inter-specific breeding between DW and BW, coupled with a MAS approach based exclusively on *Psy1-A1* and *Psy1-B1* alleles, has allowed the development of BW pre-breeding lines with enhanced grain carotenoid content (16–23% mean higher carotenoid content). This constitutes an interesting advance for wheat biofortification, considering that only two genes were subjected to selection. This approach offers new opportunities towards the development of new common wheat-derived products with a golden colour, as achieved in the new cereal crop tritordeum. Further evaluation of the agronomic performance should be performed in the future at several locations in order to determine their potential to become new varieties. In any case, they can be used as recurrent parents in BW breeding programs for carotenoid biofortification.

## Figures and Tables

**Table 1:** Genotypic characterization for *Psy1-A1* and *Psy1-B1* and carotenoid composition of the parental lines.

Genotype ^1^	YP7A2 ^2^	YP7B4 ^2^	Zeax ^3^	FLut ^4^	LutME ^5^	LutDE ^6^	βCar ^7^	TCar ^8^
‘Kiko Nick’ (DW)	1001	1	0.133	1.572	0.019	0.000	0.037	1.761
12THES4515 (BW)	1684	0	0.144	0.479	0.190	0.158	0.007	0.978
ES07H044SWC29 (BW)	1684	0	0.119	1.804	0.029	0.000	0.014	1.966

^1^ DW = durum wheat, BW = bread wheat; ^2^ For YP7A2, the fragments amplified in bp are indicated. For YP7B4, 1 = presence, 0 = absence. DW alleles are associated with higher carotenoid content; ^3^ Zeax = Zeaxanthin; ^4^ FLut = Free Lutein = (*all-E*)-Lutein + (*Z*)-Lutein isomers; ^5^ LutME = Lutein monoesters; ^6^ LutDE = Lutein diesters; ^7^ βCar = β-Carotene; ^8^ TCar = Total carotenoids = Zeax + FLut + LutME + LutDE + βCar.

**Table 2:** Mean carotenoid composition of progenies contrasting for *Psy1-A1* and *Psy1-B1* alleles.

Cross/(Alleles) ^1^	Zeax ^2^	FLut ^3^	LutME ^4^	LutDE ^5^	βCar ^6^	TCar ^7^
KN × C29 (DW)	0.187	2.267	0	0	0.029	2.483
KN × C29 (BW)	0.183	1.802	0	0	0.029	2.014
KN × THES (DW)	0.318	2.291	0.442	0.097	0.066	3.213
KN × THES (BW)	0.233	2.057	0.345	0.065	0.050	2.751

^1^ Each progeny is identified by the cross, where KN × C29 = ‘Kiko Nick’ × ES07H044SWC29 and KN × THES = ‘Kiko Nick’ × 12THES4515. Progenies carrying alleles from durum wheat for the genes *Psy1-A1* and *Psy1-B1* are identified as (DW), while those carrying the alleles from bread wheat are identified as (BW); ^2^ Zeax = Zeaxanthin; ^3^ FLut = Free Lutein = (all-*E*)-Lutein + (*Z*)-Lutein isomers; ^4^ LutME = Lutein monoesters; ^5^ LutDE = Lutein diesters; ^6^ βCar = β-Carotene; ^7^ TCar = Total carotenoids = Zeax + FLut + LutME + LutDE + βCar.

## Data Availability

Data are contained within the article and supplementary materials.

## Supplementary Materials

The following supporting information can be downloaded at: https://www.mdpi.com/article/10.3390/foods12071365/s1, Table S1. Detailed carotenoid composition of the parental lines used in this work; Table S2. Detailed carotenoid composition and genotypic constitution for markers YP7A-2 and YP7B-4 of the BW lines obtained in this work through inter-specific hybridization.
